# Ensemble machine learning method for δ^18^O prediction in groundwater

**DOI:** 10.1038/s41598-026-57131-y

**Published:** 2026-06-21

**Authors:** Faten. A. Mohamed, Mostafa Sadek, Nema Mohamed Kandil

**Affiliations:** Nuclear and Radiological Safety Research Center (NRSRC), Egyptian Atomic Energy Authority (EAEA), Cairo, Egypt

**Keywords:** RFR, GBR, δ^18^O variability prediction, Groundwater management, Egypt, Environmental sciences, Hydrology

## Abstract

Groundwater δ^18^O interpretation in arid regions is challenging due to complex recharge, salinization, and limited datasets. We applied ensemble machine learning, Random Forest, Lasso, and Gradient Boosting, to predict δ^18^O using hydrochemical and spatial variables in the mid-Nile Valley. Under random splitting, RFR performed best (R^2^ = 0.83), followed by Lasso (0.81) and GBR (0.80). Longitude, chloride, potassium, and magnesium emerged as the main predictors, consistent with hydrochemical evidence. But performance dropped under spatial cross-validation, a more realistic test: RFR R^2^ = 0.50, Lasso = 0.45, GBR = 0.41. So, part of the predictive skill comes from spatial structure, not transferable relationships. A sensitivity test without longitude showed that hydrochemical variables alone explain about 40% of the δ^18^O variance (R^2^ = 0.405). Within the data-rich range (-3‰ to -1‰, *n* = 54), the model achieved a mean absolute residual of 0.70‰ and a 95% prediction interval of ± 2.26‰. Outside this range, for Eocene end-members (*n* = 7) and Quaternary enriched samples (*n* ≈ 6), uncertainty increased substantially. Machine learning is useful for isotope prediction in data-scarce regions, but results must be interpreted with caution. Spatial effects matter. The model is best suited for site-specific analysis within its reliable range. Future work should focus on collecting more end-member samples to bridge the isotopic gap.

**Supplementary Information:**

The online version contains supplementary material available at 10.1038/s41598-026-57131-y.

## Introduction

Groundwater is the largest accessible freshwater reservoir on Earth, supplying nearly half of global drinking water and approximately 40% of irrigation demand. More than 2.5 billion people depend on it for daily needs, making its sustainable management a central challenge under pressures from population growth, agricultural intensification, and climate variability^1,2^. Beyond its socioeconomic role, groundwater sustains ecosystems and provides resilience in climate adaptation strategies^3^.

Stable isotopes of oxygen (δ^18^O) and hydrogen (δ^2^H) are established tracers for identifying recharge sources, flow paths, and mixing processes within the hydrological cycle. They provide diagnostic information on aquifer connectivity, seawater intrusion, and evaporative enrichment that cannot be obtained from conventional hydrochemical methods^4–7^. Despite their proven utility, isotope applications remain constrained by analytical costs and sparse monitoring networks, particularly in arid and semi-arid regions.

Prediction of δ^18^O concentrations from groundwater chemistry is inherently complex, as isotopic composition reflects the combined effects of climate variability, geological controls, and hydrochemical processes. These interactions are often nonlinear and spatially heterogeneous, making it difficult to capture them using conventional approaches. Geostatistical methods such as ordinary kriging, regression kriging, and cokriging have been widely used to interpolate isotope data^8,9^. While these techniques are effective in preserving spatial continuity, their performance may be limited in areas with strong heterogeneity or complex hydrogeological settings, where assumptions of linearity and stationarity are not fully satisfied^10^.

Linear regression approaches have traditionally been used as baseline models in hydrogeological studies due to their simplicity and interpretability^11–14^. However, classical multiple linear regression often struggles in the presence of multicollinearity and nonlinear relationships among predictors^15^. To address these limitations, regularized regression methods such as LASSO (Least Absolute Shrinkage and Selection Operator) have been increasingly adopted. LASSO introduces a penalty term that constrains model coefficients, improving stability and enabling automatic variable selection, which is particularly useful when dealing with correlated variables and relatively small datasets^16^.

In recent years, machine learning (ML) approaches have gained increasing attention in hydrogeology because of their ability to capture nonlinear relationships and complex interactions among environmental variables. Ensemble learning techniques, including bagging and boosting, further enhance predictive performance by combining multiple models and reducing variance^17^. Among these, Random Forest (RF) and Gradient Boosting Machines (GBM) have been widely applied in hydrological and geochemical studies. RF improves robustness by aggregating predictions from multiple decision trees trained on bootstrapped samples^18^, while GBM iteratively minimizes residual errors and can achieve high accuracy when properly tuned^19,20^. These methods have shown strong performance in applications such as groundwater quality assessment, geochemical mapping, and hydrochemical prediction, highlighting their ability to represent complex environmental processes^21–23^.

Recent advances have seen machine learning increasingly applied to the modeling of stable isotopes, particularly in precipitation systems. By integrating environmental variables and, in some cases, remote sensing data, these approaches have improved the prediction of δ^18^O and δ^2^H across a range of hydroclimatic settings^24–28^. Compared with traditional regression methods, ensemble models are often better at capturing spatial variability, which is especially valuable in regions where monitoring data are sparse or unevenly distributed.

Although most of this work has focused on precipitation, there is growing evidence that similar approaches can be applied to groundwater systems. Early studies suggest that machine learning can support isotope prediction and interpretation in groundwater systems, but challenges remain. In particular, spatial dependence, limited sample sizes, and questions around model transferability continue to constrain broader application^29,30^.

Stable oxygen isotopes (δ^18^O) play a central role in hydrogeological investigations, as they provide insight into recharge sources, mixing processes, and the influence of climatic conditions^31^. Their spatial and temporal variability reflects the combined effects of climate, topography, and geological structure, making prediction inherently complex. While geostatistical approaches offer strong spatial consistency, machine learning models often provide greater flexibility and predictive power. Combining these perspectives holds clear potential, but issues such as uncertainty quantification and applicability across different aquifer systems remain insufficiently addressed.

In this context, the objective of the present study is to develop an ensemble-based modeling framework for predicting groundwater δ^18^O concentrations using readily available hydrochemical data. The approach is designed to retain physical interpretability while extending beyond conventional interpolation methods. By integrating tree-based ensemble models with feature importance analysis and multiple validation strategies, the study aims to provide a practical tool for investigating recharge dynamics, aquifer mixing, and hydrochemical evolution in data-limited and spatially heterogeneous environments.

## Study area and its characteristics

Egypt is one of the most water-scarce countries, with most freshwater resources derived externally through Nile River discharge. Rainfall is negligible, and groundwater reserves are limited. Pressures from population growth, climate variability, and upstream dam construction in the Upper Nile Basin further constrain water availability. Effective resource development requires management strategies that balance supply and demand while minimizing local socioeconomic impacts.

**Fig. 1:**
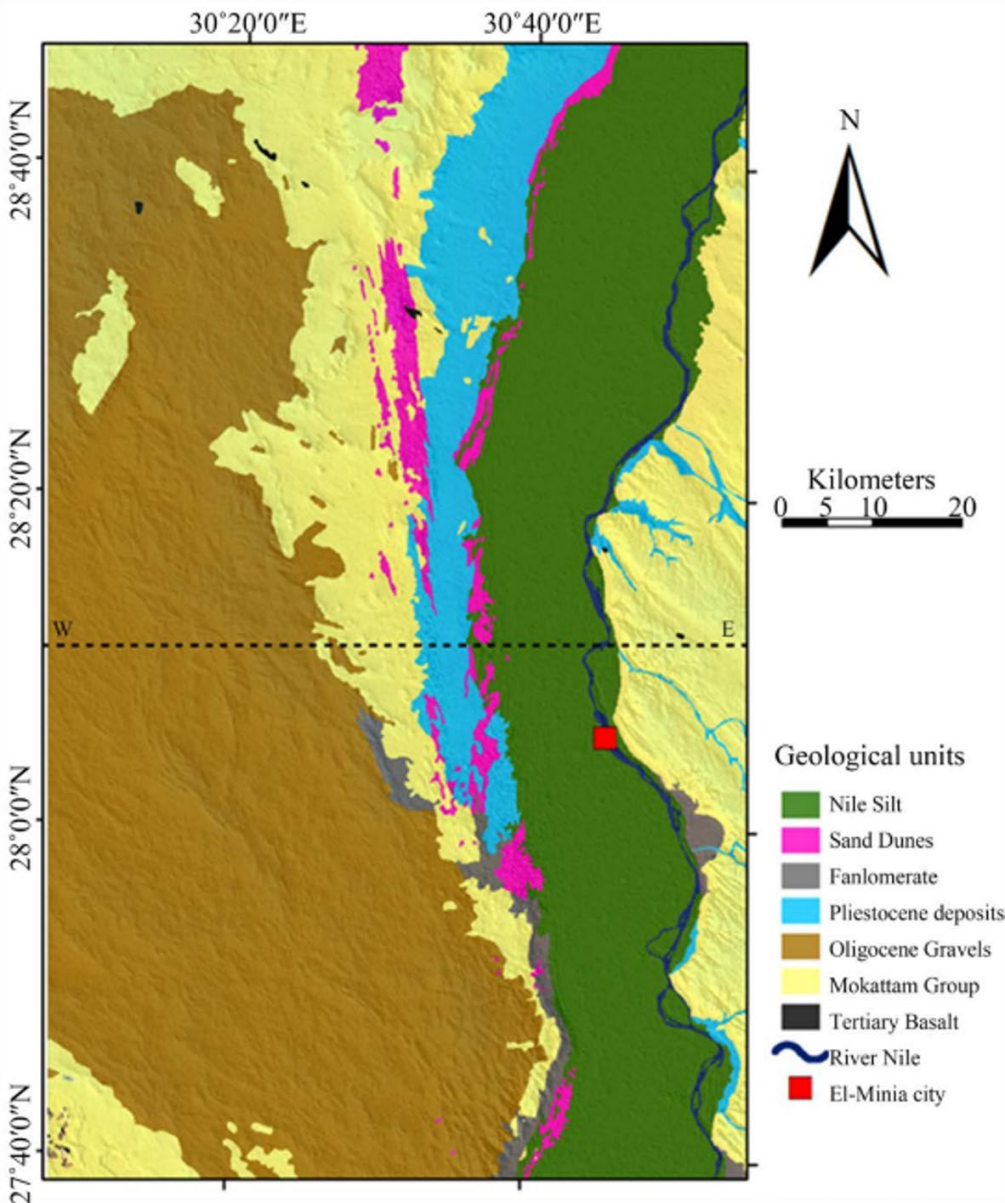
Geological map of the El-Minia region^32^.

The study area lies along the western desert margins of the mid-Nile Valley. The climate is arid, with less than 15 mm of annual rainfall. Summers average 40 °C, while winters average 20 °C. Three geomorphological units extend east–west across the region. The present alluvial plain, adjacent to the Nile River, consists of Holocene silt and clay deposits. The old alluvial plain is composed of Pleistocene sands and gravels and includes reclaimed desert lands and scattered urban zones. The westernmost unit, the Lower Eocene Plateau, is moderately elevated and formed of structurally controlled limestone overlain by sandy and gravelly alluvial deposits. These geomorphological features influence groundwater flow and recharge dynamics (Fig. 1).

**Fig. 2:**
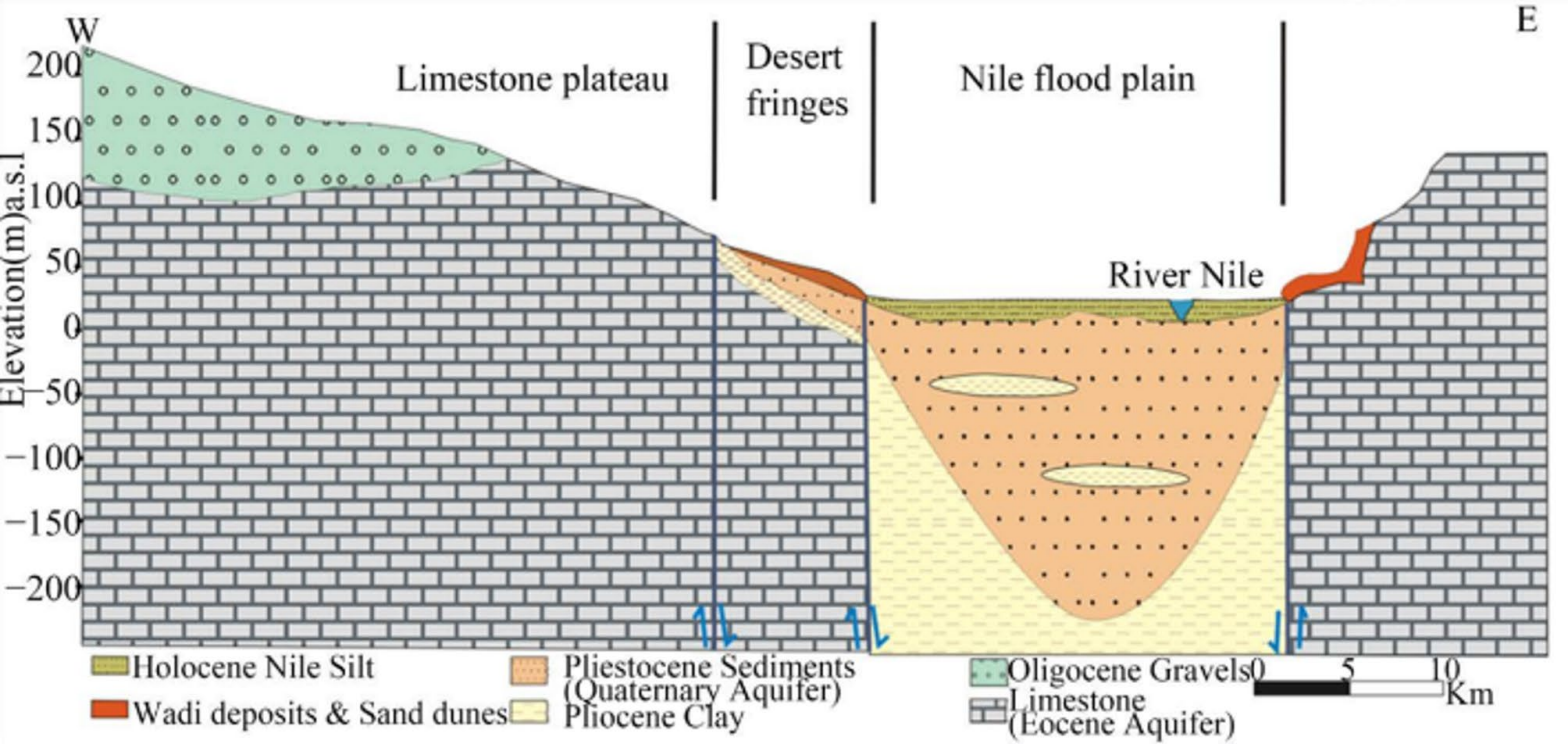
W–S hydrogeological cross-section (AA′) illustrating aquifer systems and structural features, Adapted from Asmoay^33^, originally based on RIGW^34^, and reused under the Creative Commons Attribution 4.0 International License (CC BY 4.0).

From a hydrogeological perspective, the area hosts a multi-layered aquifer system extending from the Nile floodplain eastward to the Eocene plateau (Fig. 2). The Quaternary aquifer is shallow and unconfined, consisting of coarse sand and gravel interbedded with clay lenses, overlain by Holocene Nile silt and sandy clay that form a semi-permeable to impermeable cap. Thickness varies from zero at the desert fringes to about 16 m near the Nile, and recharge occurs mainly through seepage from irrigation canals and infiltration of return flows. In the northwest, the Oligocene aquifer, composed of the Qatrani and Katkutt formations, functions as a shallow unconfined system of sand and gravel. The Middle Eocene limestone aquifer, the principal aquifer under investigation, consists of Nummulitic and Dolomitic limestone with interbedded marl and shale. It is unconfined to semi-confined and exhibits high hydraulic conductivity due to fracturing, jointing, and karstic features. Recharge is mixed, receiving water from the Quaternary aquifer and paleowater migrating upward through fault zones. Beneath these units lies the Samalut aquifer, composed of hard, fossiliferous limestone. It is hydraulically connected to the overlying formations and shows moderate variability in groundwater levels. At greater depths, the Nubian Sandstone aquifer system comprises the Maastrichtian Khoman Chalk, Campanian Abo Roash, and Lower Cenomanian Bahariya formations. This aquifer contains paleowater and is not subject to modern recharge, although upward leakage may occur through faults and fractures.

A west–east geological cross-section (AA′) illustrates the stratigraphic configuration and inferred fault zones that facilitate vertical hydraulic connectivity. Groundwater level variations across the Oligocene and Samalut aquifers provide insights into flow dynamics along the geomorphological gradient.

## Materials and methods

**Fig. 3:**
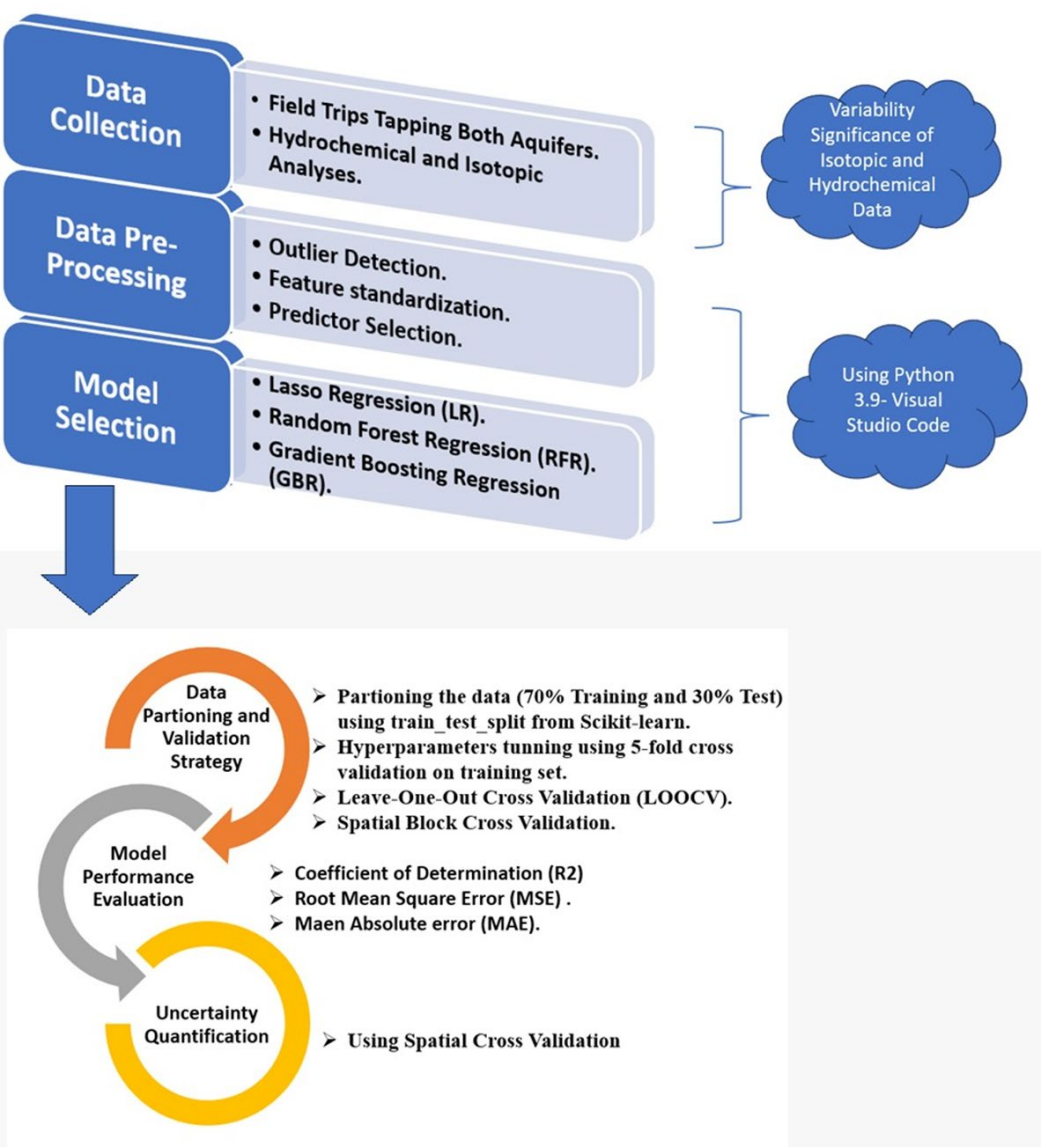
Methodological flowchart outlines the sequential steps: data collection, preprocessing, feature selection, model training, validation, for δ^18^O prediction in groundwater.

To systematically evaluate the δ^18^O prediction in groundwater, a multi- stage workflow was developed as illustrated in Fig. 3.

### Field and laboratory work

**Fig. 4:**
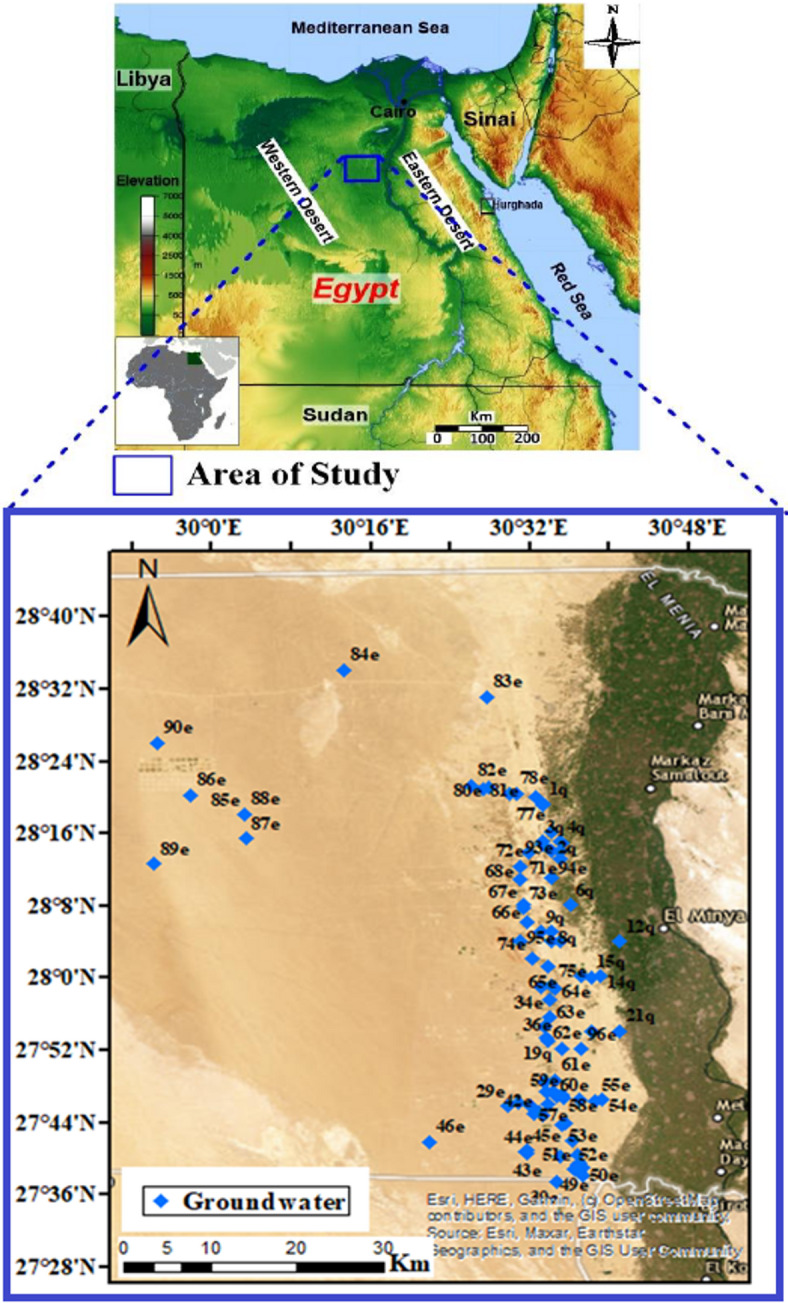
Location map of the study area in Egypt, showing groundwater sampling wells. The base map consists of satellite imagery downloaded from the **USGS Earth Explorer** (https://earthexplorer.usgs.gov) and processed in **QGIS** (version 3.28; QGIS Association, https://qgis.org).

A field campaign was conducted in March 2024 to collect groundwater samples from the Eocene and Quaternary aquifers. In total, 100 samples were obtained, with in situ measurements of coordinates, electrical conductivity (EC), pH, and depth recorded at each site (Fig. 4). Major ions, including Na^+^, Ca^2+^, Mg^2+^, K^+^, Cl^–^, SO_4_^2–^, and HCO_2_^–^, were analysed following standardized protocols^35^, and concentrations were reported in milligrams per liter (mg/L). Environmental isotopes (δ^18^O and δ^2^H) were measured using Laser Spectroscopy (Picarro Model 2120i). Isotope values were expressed in per mil (‰) deviation from Vienna Standard Mean Ocean Water (VSMOW), using the conventional δ notation:

1 $$\delta \left( \permil \right) = \left( {\frac{{R_{{sample}} }}{{R_{{standard}} }} - 1} \right) \times 1000$$

where ${R_{{\mathrm{sample}}}}$and ${R_{{\mathrm{standard}}}}~$represent the isotopic ratios (^18^O/^16^O and ^2^H/^1^H) of the sample and reference material, respectively. All analyses were performed at the Central Laboratory of Environmental Isotope Hydrology, Egyptian Atomic Energy Authority.

### Data pre-processing

Outliers were identified using the Mahalanobis Distance (MD), which accounts for the multivariate structure of the dataset by incorporating correlations among predictor variables. Observations exceeding the chi-square critical value at the 95% confidence level were considered statistically anomalous and removed from the dataset (four samples in total)^36^. This step was undertaken to improve data consistency and reduce the influence of extreme values on model performance.

To ensure transparency, a sensitivity analysis was also performed by repeating the modeling workflow with and without outlier removal, allowing the impact of this step on predictive performance to be explicitly evaluated.

Following outlier treatment, all input variables were standardized using z-score normalization:

2 $${x_{{\mathrm{scaled}}}}=\frac{{{X_i} - \mu }}{{{\sigma _X}}}$$

where ${X_i}$is the raw input value, $\mu$is the feature mean, and ${\sigma _X}$is the standard deviation of the variable. Standardization was applied to ensure comparability across predictors and to support regularized regression methods such as LASSO. Although tree-based models (e.g., Random Forest and Gradient Boosting) are generally scale-invariant, a consistent preprocessing workflow was maintained across all models (Tables 1, 2).

Pearson correlation analysis was used as an initial screening step to identify variables with meaningful relationships to δ^18^O (|r| ≥ 0.4, *p* ≤ 0.01; Table 3). The resulting correlation structure revealed strong interdependencies among several hydrochemical parameters, including EC, Na^+^, Cl^–^, Mg^2+^, Ca^2+^, and SO_4_^2–^, reflecting shared salinity and mineralization processes.

These interrelationships indicate the presence of multicollinearity, which is typical in hydrochemical datasets. Rather than attempting to remove collinearity entirely through variable selection, this study addressed it through a combination of correlation-based screening and model choice. Ensemble methods (RFR and GBR) are relatively robust to correlated inputs, while LASSO provides additional regularization by shrinking less informative coefficients, thereby improving model stability.

### Model selection and validation strategy

Three regression models were implemented to predict δ^18^O in groundwater: LASSO regression, Random Forest Regression (RFR), and Gradient Boosting Regression (GBR). LASSO was adopted as a linear baseline model because it provides coefficient regularization and is well suited for datasets with potential multicollinearity among hydrochemical variables. In contrast, RFR and GBR were selected for their ability to capture nonlinear relationships and complex interactions, which are common in hydrogeochemical systems.

The dataset was divided into training (70%) and testing (30%) subsets using the *train_test_split* function in Scikit-learn. Model hyperparameters were optimized through cross-validation on the training set, with the best configurations selected based on mean R^2^ across folds. Model performance was further evaluated using MSE and MAE to ensure consistency in error representation.

Given the relatively limited sample size and the heterogeneous nature of the study area, additional validation strategies were implemented to better assess model robustness. Leave-One-Out Cross-Validation (LOOCV) was applied using the optimized models to evaluate sensitivity to individual observations. This approach was considered appropriate because each sample represents a distinct hydrochemical condition, and the influence of single observations may be significant in a small dataset^37^.

To address spatial dependence within the dataset, a spatially structured cross-validation scheme was also implemented using grouped folds based on longitudinal blocks. This approach reduces the likelihood of spatial information leakage between training and testing subsets and provides a more realistic assessment of model generalizability across different parts of the study area.

**Table 1:** Best regression hyperparameters for small datasets.

Parameter	Recommended range	Theoretical Justification
n. estimators	50–100	Diminishing returns beyond 100 trees for generalization error^19^
max_- depth	2–4	Restricts tree complexity to reduce overfitting in small samples^38^
Learning-rate (GBR)	0.01–0.1	Based on the shrinkage principle for improved generalization in gradient boosting^20^

### Model evaluation metrics and uncertainty

Model performance was assessed using the coefficient of determination (R^2^), mean square error (MSE), and mean absolute error (MAE), defined as:

3 $${R^2}=1 - \frac{{\sum {{({{\hat {y}}_i} - {y_i})}^2}}}{{\sum {{({y_i} - \mathop y\limits^{\prime } )}^2}}}$$

4 $$MSE=\frac{{\sum {{({{\hat {y}}_i} - {y_i})}^2}}}{n}$$

5 $$MAE=\frac{{\sum \mid {{\hat {y}}_i} - {y_i}\mid }}{n}$$

where ${y_i}$and ${\hat {y}_i}$ denote observed and predicted values, $\mathop y\limits^{\prime } ~$is the mean of observed values, and *n* is the sample size. Model generalization was evaluated using both LOOCV and spatial cross-validation, as described above, to capture different aspects of predictive performance. Uncertainty was quantified using the variability of predictions across individual trees within the Random Forest model. For each prediction, the standard deviation of tree outputs was used to estimate uncertainty, and approximate 95% prediction intervals were calculated as:

6 $$\hat{y}_{i} \pm {\mathrm{1}}.{\mathrm{96}} \times \sigma$$

To avoid over-optimistic estimates, uncertainty analysis was interpreted primarily in the context of spatial cross-validation rather than random resampling, providing a more conservative assessment of model reliability under spatially independent conditions.

### Computational environment and reproducibility

All analyses were conducted in Python 3.9 using Visual Studio Code (version 1.109). Core libraries included NumPy and Pandas for data handling, Scikit-learn for model development and validation, SciPy for statistical analysis (including Mahalanobis distance), and Matplotlib and Seaborn for visualization^39^.

## Results and discussion

### Variability and significance of hydrochemical and isotopic data

Table 2 summarizes the descriptive statistics for major ions and total dissolved solids (TDS) in the groundwater samples. The wide concentration ranges and high coefficients of variation indicate significant hydrochemical heterogeneity. This variability arises from multiple geochemical processes, including salinization through rock–water interactions and mixing of recharge waters with distinct chemical compositions.

The variability in solute concentrations and isotopic signatures illustrates the influence of mineral dissolution, evaporative concentration, and mixing of distinct recharge sources. Crucially, the heterogeneity observed in major ion chemistry is mirrored in δ^18^O and δ^2^H values, as both reflect the same recharge pathways and geochemical processes. This coupling provides a direct framework for interpreting isotopic dynamics within the aquifer system.

**Table 2:** Descriptive statistics of TDS and major ions content.

Eocene ground water samples						Quaternary groundwater samples				
Variables	Min	Max	Mean	Std. Dev	coff. var	Min	Max	Mean	Std. Dev	coff. Var
TDS	436.00	6422.66	1443.94	1193.65	0.83	346.21	4279.02	1881.07	1435.79	0.76
Na	40.00	1553.31	280.42	274.86	0.98	33.68	722.38	266.86	239.35	0.90
K	3.12	26.00	10.33	6.70	0.65	-0.03	22.02	7.91	6.59	0.83
Mg	24.00	144.00	61.31	33.71	0.55	4.80	163.20	41.39	44.33	1.07
Ca	10.00	272.00	59.43	53.38	0.90	35.00	320.00	143.94	95.60	0.66
Cl	65.00	1834.64	454.80	408.70	0.90	26.98	1544.61	413.51	473.48	1.15
SO_4_	24.60	1927.25	180.07	318.27	1.77	31.72	1473.95	381.90	444.01	1.16
HCO_3_	55.00	390.00	214.37	55.76	0.26	65.76	304.88	173.23	55.93	0.32
δ ^18^O	-10.54	1.62	-2.88	2.71	-0.94	-3.09	3.45	0.08	2.39	−

### Isotopic mixing and recharge sources

**Fig. 5:**
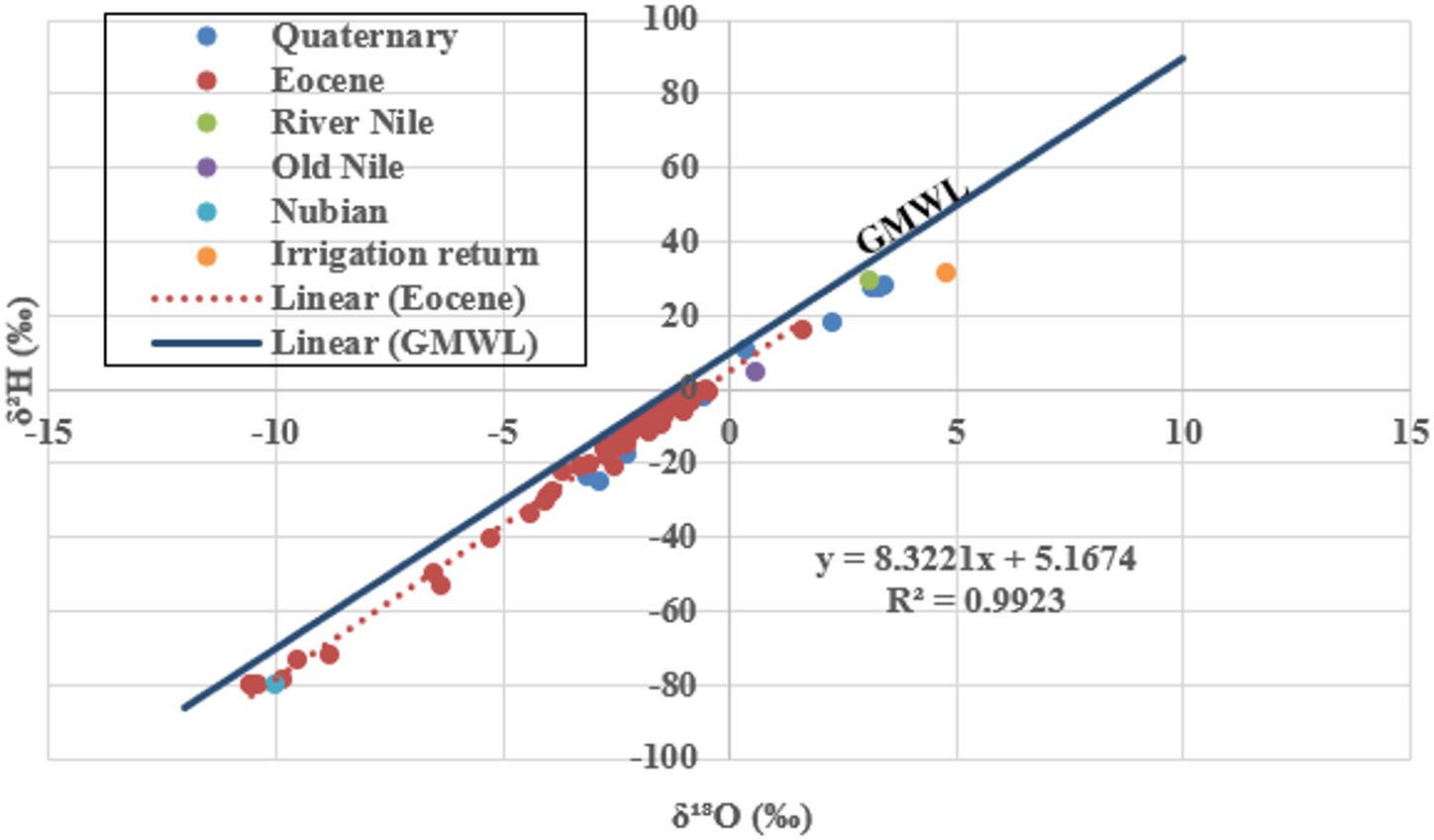
δ^2^H versus δ^18^O relationship in groundwater samples, showing isotopic signatures across different water sources. The dashed line represents the regression line for Eocene samples, while the solid line corresponds to the Global Meteoric Water Line (GMWL)^40^.

Measured δ^18^O values in groundwater samples ranged from − 10.18‰ to + 2.88‰, while δ^2^H values ranged from − 78.48‰ to + 25.29‰. These ranges encompass the isotopic signatures of Egypt’s two primary recharge sources: modern Nile River water and deep-seated paleowater stored in the Nubian Sandstone Aquifer. The δ^2^H–δ^18^O relationship (Fig. 5) indicates that the Eocene limestone aquifer receives recharge from both sources, with isotopic compositions reflecting mixing between recent infiltration and upward leakage from deeper formations.

To estimate the relative contribution of paleowater, a two-endmember mixing model was applied using the following equation:

7 $$\frac{{{Q_{{\mathrm{old}}}}}}{{{Q_{{\mathrm{total}}}}}}=\frac{{{\delta ^{18}}{O_{{\mathrm{total}}}} - {\delta ^{18}}{O_{{\mathrm{new}}}}}}{{{\delta ^{18}}{O_{{\mathrm{old}}}} - {\delta ^{18}}{O_{{\mathrm{new}}}}}}$$

Here, ${Q_{{\mathrm{old}}}}/{Q_{{\mathrm{total}}}}$ represents the proportion of Nubian aquifer water in the sample, ${\delta ^{18}}{O_{{\mathrm{total}}}}$ is the measured isotopic value, ${\delta ^{18}}{O_{{\mathrm{old}}}}$ corresponds to the Nubian Sandstone endmember, and ${\delta ^{18}}{O_{{\mathrm{new}}}}$ reflects the Nile water signature. The contribution from Nile water is calculated as $1 - {Q_{{\mathrm{old}}}}/{Q_{{\mathrm{total}}}}.$

**Fig. 6:**
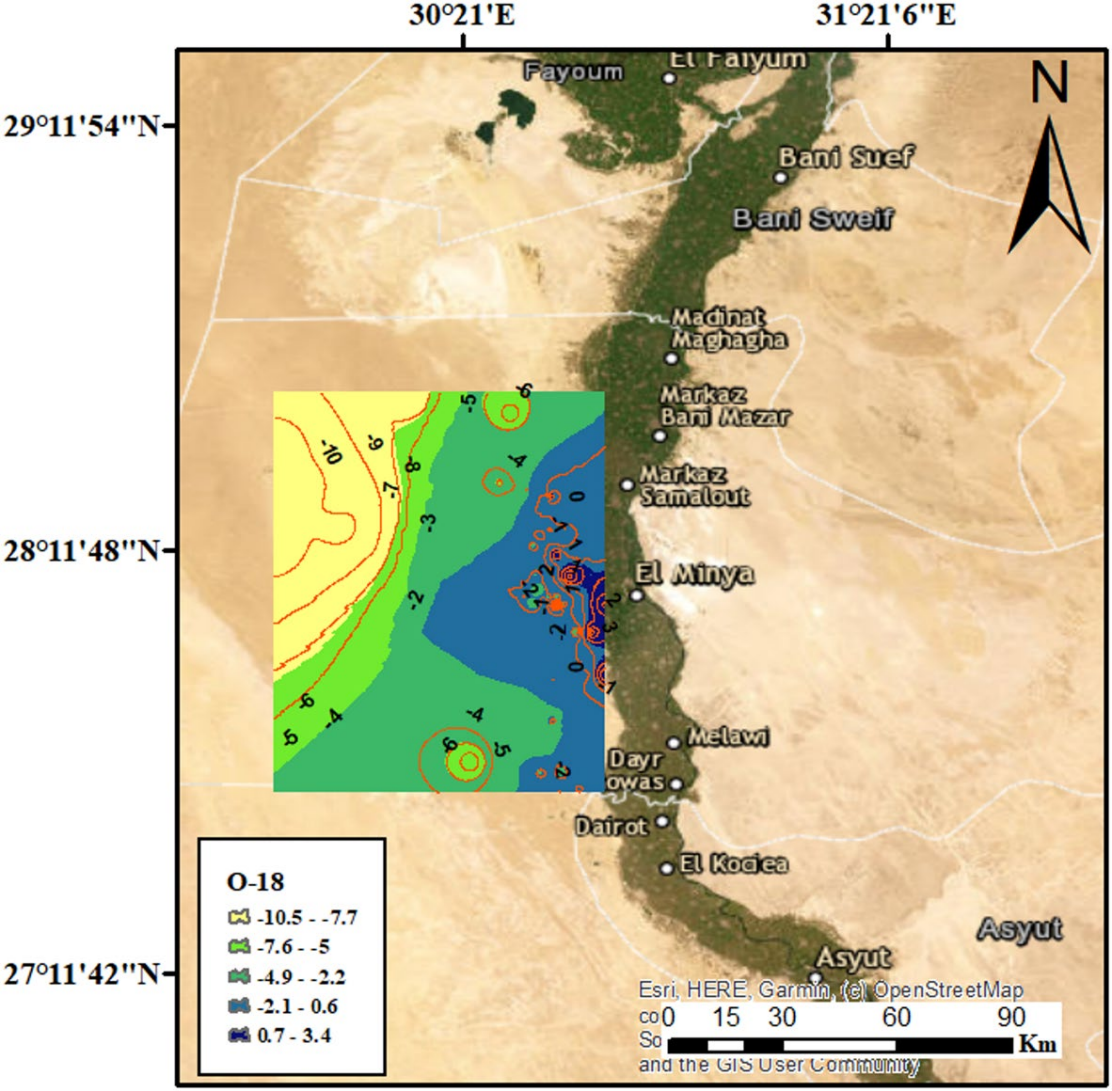
Spatial distribution of δ^18^O concentrations in groundwater across the study area, interpolated using the Inverse Distance Weighting (IDW) method^41,42^.

Results show substantial spatial variation in recharge composition. In areas adjacent to the Nile floodplain, modern water dominates, with Nile contributions reaching up to 99%. In contrast, distal zones on the elevated limestone plateau show minimal influence from recent recharge, with Nile water accounting for as little as 0.2%. The spatial distribution of δ^18^O values (Fig. 6) reveals a clear east–west gradient: modern recharge is concentrated near the river, while depleted isotopic signatures westward indicate paleowater dominance beyond 40–50 km from the floodplain.

**Fig. 7:**
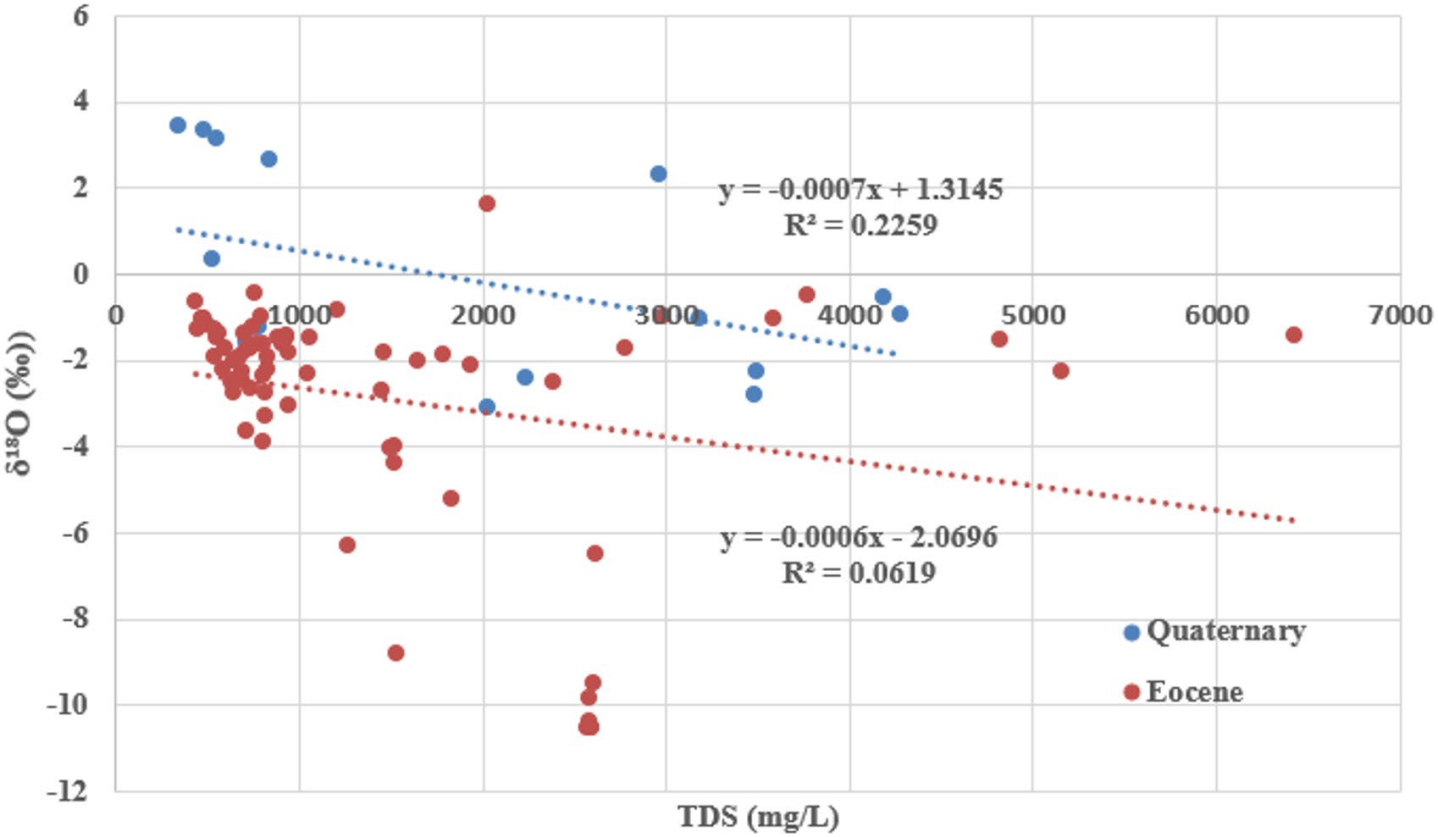
Scatter plot showing the relationship between δ^18^O (‰) and total dissolved solids (TDS, mg/L) in groundwater samples from Quaternary and Eocene aquifers. The plot reveals distinct isotopic and salinity trends, with Eocene samples generally exhibiting higher TDS and more depleted δ^18^O values, suggesting deeper circulation and prolonged water–rock interaction.

The relationship between δ^18^O and total dissolved solids (TDS) was further examined to explore links between isotopic composition and salinity (Fig. 7). In Quaternary aquifer samples, a weak negative correlation was observed (R^2^ = 0.227), while the Eocene samples showed an even weaker association (R^2^ = 0.062). These low coefficients confirm that δ^18^O alone does not explain salinity variability. The scattered pattern reflects the influence of multiple overlapping processes. These include mixing between deep saline and shallow freshwater zones, mineral dissolution during water–rock interaction, and salt accumulation from evaporation, particularly in irrigated areas. Because these processes interact in nonlinear ways, simple regression models are insufficient to capture the underlying dynamics. To address this, ensemble machine learning models were employed to better represent the multivariate relationships governing isotopic behavior under variable hydrochemical conditions.

### Hydrochemical controls and rationale for model selection

The hydrochemical dataset reveals a complex groundwater system shaped by multiple overlapping processes. The scatter in δ^18^O relative to salinity indicators, along with the presence of distinct isotopic end-members, points to mixed recharge sources, evaporative enrichment, and water–rock interaction. This complexity limits simple linear interpretations. Isotopic composition is not controlled by individual solutes in a direct way. Instead, it reflects integrated hydrogeochemical processes.

Under these conditions, conventional statistical methods often miss the underlying structure of the system. Machine learning offers a useful alternative. It can capture nonlinear relationships and interactions among variables without requiring strong assumptions about process form. In this study, we combined Lasso regression with ensemble learning models. This allowed us to distinguish between dominant linear trends and more complex patterns in the data.

### Model performance evaluation

To evaluate the predictive capability of different approaches, we implemented three regression models: Lasso (linear baseline), Random Forest (RFR), and Gradient Boosting (GBR). All models used the same predictor set: longitude, Cl, Mg, and K.

Lasso regression was adopted as a baseline to examine how much δ^18^O variability can be explained through linear relationships while accounting for multicollinearity. The L1 regularization stabilizes coefficient estimates and performs implicit variable selection a useful property for hydrochemical datasets where predictors are often correlated.

The strength of this correlation structure is shown in Table 3. Pearson correlation analysis reveals a strong positive relationship between δ^18^O and longitude (*r* = 0.88), alongside moderate negative correlations with potassium (*r* = − 0.47), magnesium (*r* = − 0.67), and chloride (*r* = − 0.49). These correlated variables were therefore retained as predictors. It is important to note that these hydrochemical parameters are not direct controls on isotopic composition. Rather, they co-vary with broader processes such as salinization, evaporation, and water–rock interaction. The presence of these correlations justifies the use of Lasso, which is designed to handle exactly such interdependencies among predictors.

**Table 3:** Pearson correlation coefficients.

Variables	Long	Lat	EC	δ^18^O	Na	K	Mg	Ca	Cl	SO_4_	HCO_3_
Long	1										
Lat	− 0.47^**^	1									
EC	− 0.33^**^	0.54^**^	1								
δ^18^O	0.88^**^	− 0.30^**^	− 0.29^**^	1							
Na	− 0.28^**^	0.50^**^	0.95^**^	− 0.25^*^	1						
K	− 0.61^**^	0.42^**^	0.55^**^	− 0.47^**^	0.58^**^	1					
Mg	− 0.69^**^	0.44^**^	0.76^**^	− 0.67^**^	0.67^**^	0.62^**^	1				
Ca	-0.08	0.50^**^	0.79^**^	0.00	0.68^**^	0.36^**^	0.40^**^	1			
Cl	− 0.51^**^	0.51^**^	0.95^**^	− 0.49^**^	0.88^**^	0.58^**^	0.89^**^	0.64^**^	1		
SO_4_	0.08	0.36^**^	0.70^**^	0.17	0.78^**^	0.40^**^	0.22^*^	0.76^**^	0.47^**^	1	
HCO_3_	-0.06	-0.02	− 0.27^*^	-0.098	-0.19	-0.04	-0.07	− 0.37^**^	-0.19	− 0.36^**^	1

*. Correlation is significant at the 0.05 level (2-tailed).

**. Correlation is significant at the 0.01 level (2-tailed).

Lasso achieved a test R^2^ of 0.81, with a small training–test gap (ΔR^2^ ≈ 0.03), indicating stable generalization. This shows that a substantial portion of δ^18^O variability can be captured by a regularized linear representation of the dominant spatial–geochemical gradient.

While Lasso captures the dominant linear structure, ensemble methods may capture additional nonlinear patterns. We therefore implemented Random Forest and Gradient Boosting using the same predictor set.

Random Forest achieved the best overall balance between accuracy and generalization. Training R^2^ was 0.90, test R^2^ was 0.83, with low error values (MAE = 0.67‰, MSE ≈ 1.08). The small performance gap indicates effective generalization without substantial overfitting. This reflects the strength of Random Forest in reducing variance through ensemble averaging.

Gradient Boosting showed the highest training performance (R^2^ = 0.94), but test accuracy dropped to 0.82. This larger gap suggests GBR is more sensitive to overfitting, likely due to its sequential learning structure, which can amplify noise when data are limited.

Under random validation, Lasso and RFR performed similarly, with RFR showing a modest improvement. However, these results come from random splitting, which does not account for spatial structure in the data. We then tested how well the models generalize to new locations using spatial cross-validation (Table 4).

**Table 4:** Models performance metrics:

Model	*R* ^2^ (Train)	*R* ^2^ (Test)	MAE (Train)	MAE (Test)	MSE (Train)	MSE (Test)
LR	0.78	0.81	1.04	0.80	1.97	1.2
RFR	0.90	0.83	0.66	0.67	0.89	1.08
GBR	0.94	0.82	0.43	0.74	0.31	1.15

### Cross-validation and model robustness

**Fig. 8:**
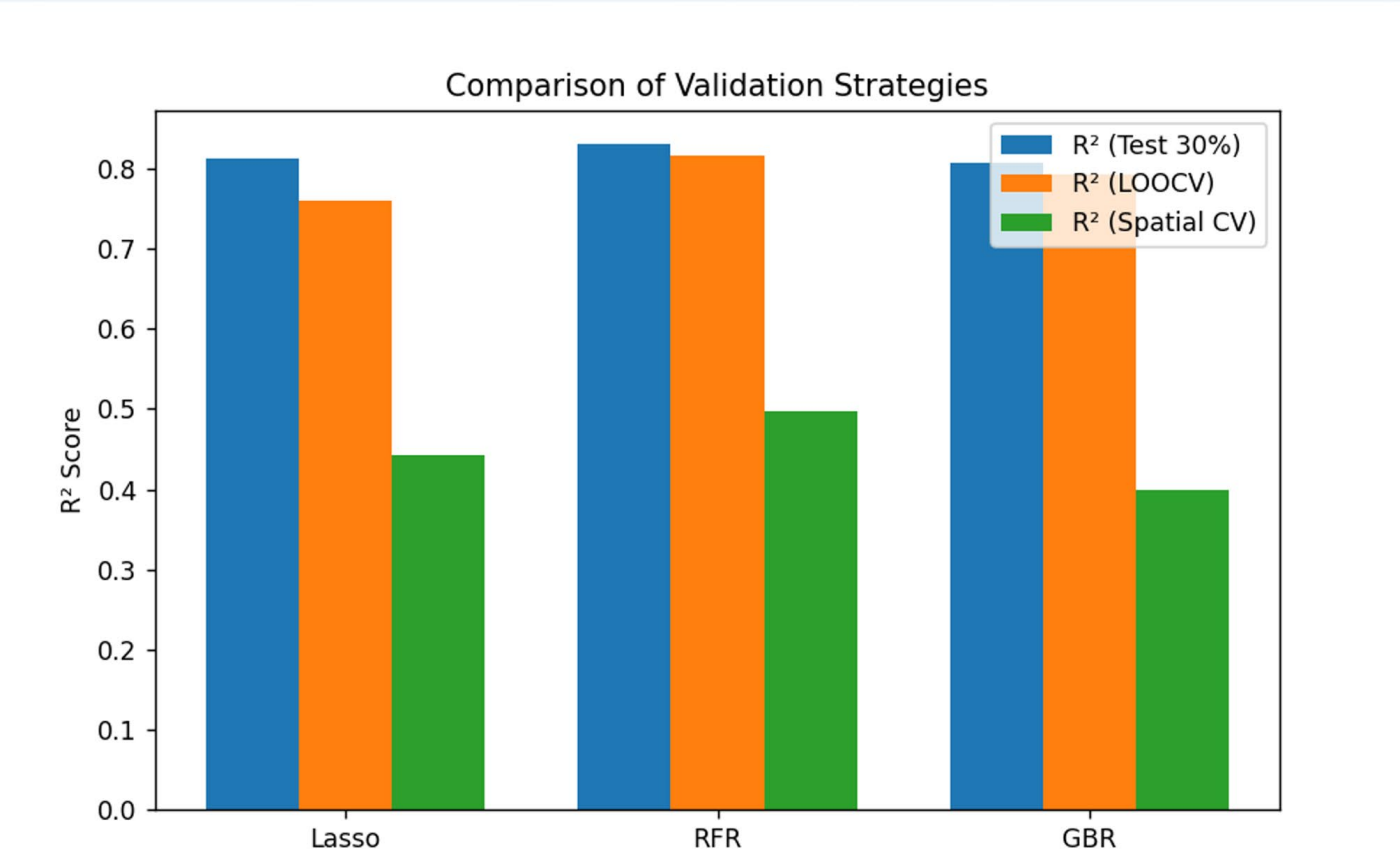
Model performance comparison. R^2^ values for Lasso, RFR, and GBR under random test split, LOOCV, and spatial cross-validation. Spatial CV gives the most realistic performance estimate.

We evaluated model stability using Leave-One-Out Cross-Validation (LOOCV) and spatial cross-validation (Spatial CV). These two approaches give complementary views of model performance (Fig. 8). LOOCV tests sensitivity to individual samples. Spatial CV tests whether models can generalize to unseen locations.

Across all models, LOOCV results are broadly consistent with the test set performance. RFR shows the closest agreement (test R^2^ = 0.83, LOOCV R^2^ = 0.82), indicating stable generalization and low sensitivity to individual observations. Lasso drops from 0.81 to 0.76, suggesting some dependence on specific samples. GBR declines from 0.80 to 0.70, reflecting greater sensitivity to small variations in the training data.

Spatial cross-validation tells a different story. All models show a substantial performance decline. R^2^ values drop to 0.45 for Lasso, 0.50 for RFR, and 0.41 for GBR. This means models trained on one spatial subset struggle to predict samples from other locations. A significant part of the predictive skill seen in random splits and LOOCV comes from spatial autocorrelation, not from transferable relationships.

Among the three, RFR holds up best under spatial validation, though its advantage over Lasso is modest (0.50 vs. 0.45). This fits with how Random Forest works — independent trees reduce variance and improve robustness. GBR suffers the most from spatial separation. Its sequential learning amplifies localized patterns that do not generalize well. Lasso, while simpler, behaves more steadily than GBR, making it a solid baseline for constrained data.

Thus, relying only on random splits or LOOCV overestimates predictive capability in spatially structured data. Spatial validation gives a more realistic picture of model transferability. These findings reinforce the need to account for spatial dependence explicitly when interpreting model performance in hydrogeochemical studies.

### Sensitivity analysis

We performed two sensitivity tests to better understand what drives the model and whether our preprocessing choices affected the results.

**Test 1: Removing longitude**.

First, we asked whether the model relies mainly on spatial location or on real hydrochemical processes. To find out, we removed longitude from the predictor set and retrained Random Forest using only Cl, Mg, and K.

Without longitude, the model still achieved an R^2^ of 0.405 under spatial cross-validation. With longitude included, it was 0.497. This means longitude contributes about 19% of the predictive power, but the chemical variables alone explain roughly 40% of the variance in δ^18^O. So, the model is not just mapping space, it is picking up meaningful geochemistry.

**Test 2: Removing outliers**.

Second, we checked whether removing four outliers (identified by Mahalanobis Distance at 95% confidence) biased our results. We reran everything with those four samples included.

With the outliers included, Random Forest spatial CV R^2^ dropped from 0.50 to 0.32. Test R^2^ stayed almost the same (0.83 vs. 0.82). This tells us the outliers were hurting spatial generalization, not inflating our performance. Removing them was the right call. Full results are in Supplementary Table S1.

Together, these sensitivity tests show two things. First, the model learns real hydrochemical patterns, not just geographic location. Second, our outlier removal improved spatial generalization without making test results look artificially good.

### Prediction uncertainty from spatial cross-validation

**Fig. 9:**
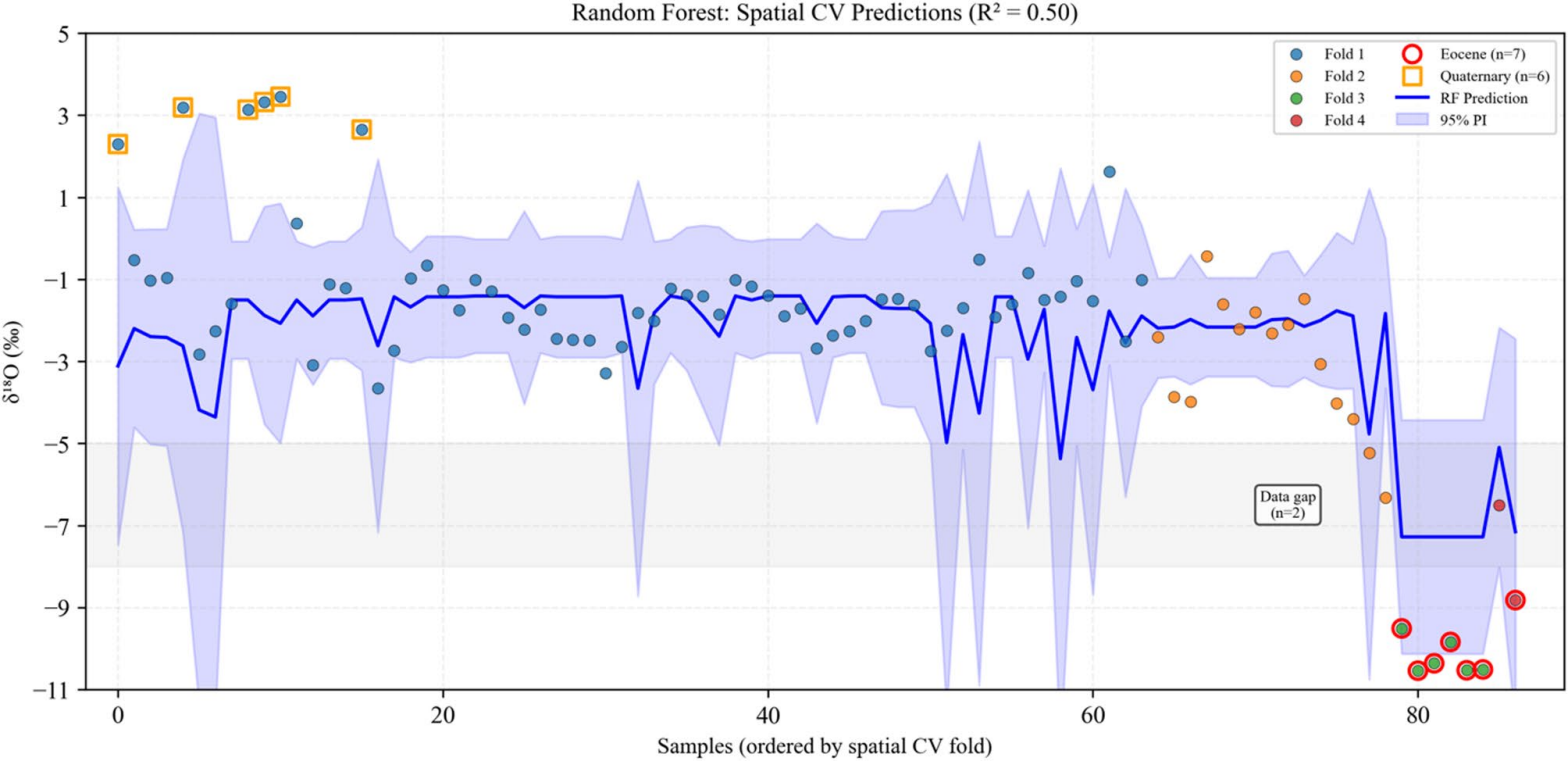
Random Forest predictions from spatial cross-validation with 95% prediction intervals. Blue line: RF prediction. Light blue band: 95% prediction interval. Colored circles: observed δ^18^O by spatial fold. Red open circles: Eocene end-members (*n* = 7). Orange squares: Quaternary end-members (*n* = 6). Gray shaded region: data gap (− 8‰ to − 5‰, *n* = 2). Spatial CV R^2^ = 0.50.

We quantified prediction uncertainty using spatial cross-validation with 4 spatial folds based on longitude bins (Fig. 9). For each test sample, we calculated uncertainty as the standard deviation of predictions across all trees in the Random Forest. This tells us how confident the model is at each location.

The 95% prediction interval (mean ± 1.96 × standard deviation) averages ± 2.42‰ across all samples (Fig. 9). Uncertainty is lowest for intermediate δ^18^O values (− 3‰ to − 1‰), where training data are most abundant (*n* = 54). Outside this range, sample sizes drop sharply. Uncertainty increases substantially for Eocene end-members (δ^18^O ≤ − 10‰, *n* = 7) and enriched samples (δ^18^O > 2.50‰, *n* ≈ 6), reflecting the limited representation of these extreme values in the training set. The widest intervals occur within the data gap between − 8‰ and − 5‰, where only two transitional samples exist.

Overall, the model is reliable for intermediate δ^18^O values (− 3‰ to − 1‰) but should not be used to predict pure Miocene or pure Quaternary end-members. The uncertainty framework provides a quantitative, spatially aware measure of prediction confidence.

### Hydrochemical evidence supporting RFR feature importance

**Fig. 10:**
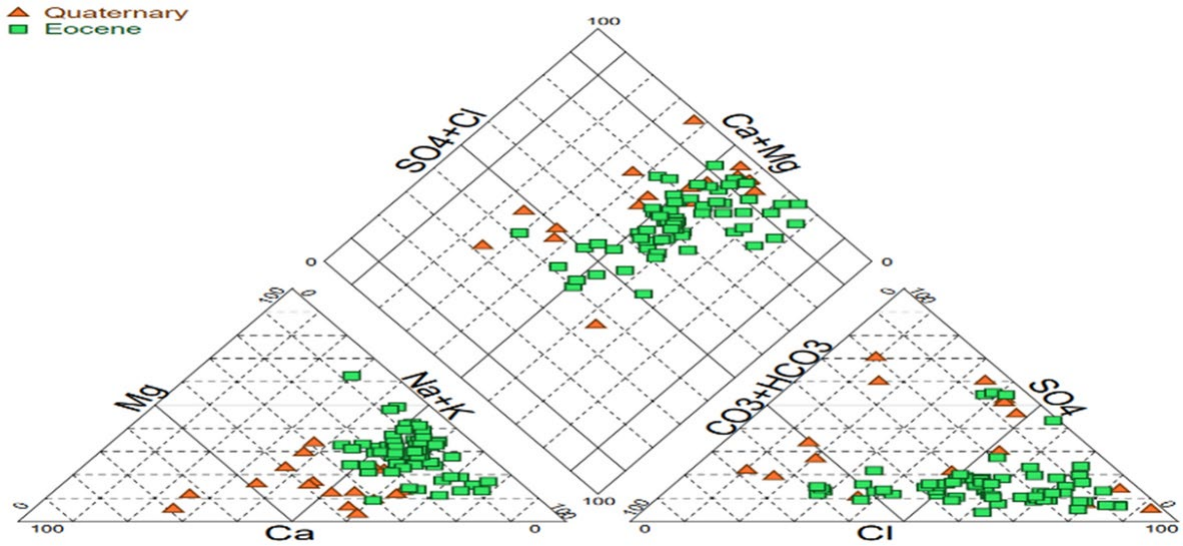
Piper diagram^43^ illustrating the major ion composition of groundwater samples from Quaternary and Eocene aquifers. The plot reveals distinct hydrochemical facies, indicating differences in water–rock interaction and recharge sources between the two systems.

Feature importance rankings from the RFR model, longitude (0.6), chloride (0.3), magnesium (0.2), and potassium (0.04), show strong correspondence with observed hydrochemical processes across the study area. Groundwater samples from the Eocene aquifer, illustrated in the Piper diagram^43^ (Fig. 10), follow two distinct hydrochemical evolution pathways: continental and marine facies. These trends reflect the leaching of terrestrial and marine salts, coupled with freshwater flushing. Saturation indices (Table 5) indicate widespread undersaturation with respect to halite, anhydrite, and gypsum, pointing to active dissolution. Calcium and magnesium saturation levels vary by aquifer: Quaternary aquifers remain undersaturated, consistent with ongoing dissolution, while Eocene aquifers are oversaturated, suggesting precipitation of calcite and dolomite.

**Table 5:** Descriptive statistics of saturation index.

Eocene groundwater samples						Quaternary groundwater samples				
Variables	Min	Max	Mean	Std. Dev	coff. Var	Min	Max	Mean	Std. Dev	coff. Var
Calcite	-1.84	1.131	0.21	0.55	2.65	-0.76	0.62	-0.33	0.35	-1.04
Dolomite	-1.99	3.58	1.87	1.07	0.57	-0.90	0.63	-0.14	0.39	-2.79
Anhydrite	-3.20	-0.76	-2.30	0.51	-0.22	-2.64	-0.64	-1.64	0.62	-0.38
Gypsum	-2.91	-0.47	-2.01	0.50	-0.25	-2.35	-0.35	-1.35	0.62	-0.46
Halite	-7.11	-4.41	-5.83	0.64	-0.11	-7.62	-4.55	-6.06	1.03	-0.17

**Fig. 11:**
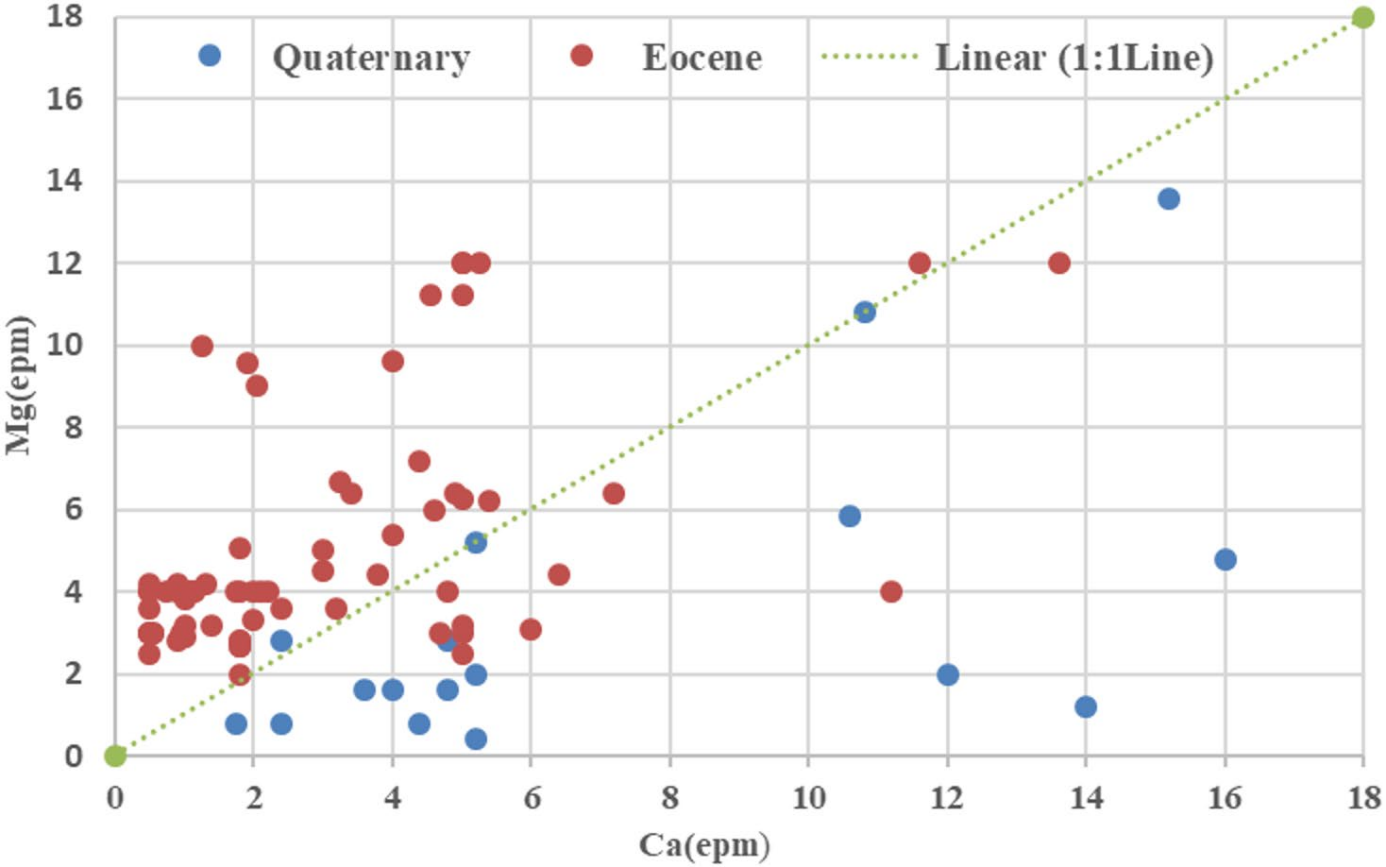
Relationship between calcium (Ca^2+^) and magnesium (Mg^2+^) concentrations (epm) in groundwater samples from Quaternary and Eocene aquifers. The 1:1 line serves as a reference for equimolar distribution. Most samples plot below the line, indicating Ca^2+^ enrichment relative to Mg^2+^. The observed patterns reflect aquifer-specific geochemical processes and potential lithological influences.

Mg/Ca ratios (Fig. 11) further differentiate aquifer behavior. In Quaternary aquifers, Mg^2+^/Ca^2+^ ratios below 1 align with calcite undersaturation and surface water recharge. In contrast, Eocene aquifers exhibit Mg^2+^/Ca^2+^ ratios greater than 1 in 93% of samples, indicating dolomite oversaturation and longer groundwater residence times^44^.

**Fig. 12:**
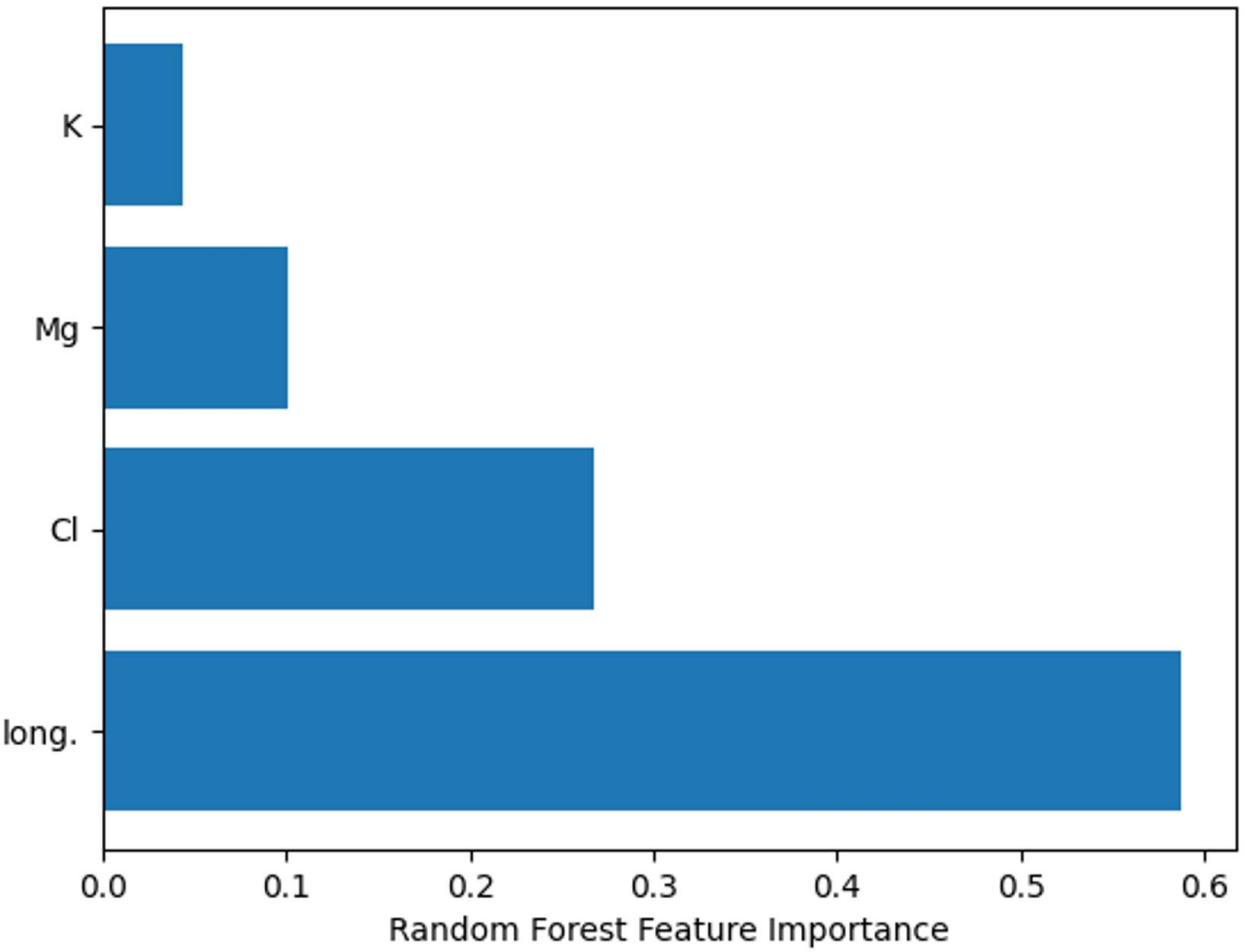
Feature importance scores from the Random Forest model predicting δ^18^O, showing longitude as the most influential variable, followed by Cl, Mg, and K.

The interpretation of feature importance (Fig. 12) reinforces these geochemical observations. Longitude captures aquifer transitions, recharge gradients, and fault-controlled flow, all of which are key drivers of δ^18^O variability. Chloride reflects salinization, evaporative enrichment, and deep mixing zones associated with δ^18^O depletion. Magnesium traces water–rock interaction and carbonate precipitation, influencing isotopic fractionation. Potassium, though less influential, signals agricultural inputs and shallow recharge modifications with limited isotopic impact.

The strong agreement between model-derived importance and independent hydrochemical evidence supports a process-based explanation of δ^18^O variability in the study area. Isotope patterns are primarily controlled by recharge source mixing, water–rock interaction, and salinization. This consistency confirms that the RFR model captures physically meaningful relationships, not just statistical correlations.

### Comparison with previous studies

Machine learning is common for precipitation isotopes but less so for groundwater, especially in complex aquifers (Table 6). Most earlier studies focused on high R^2^ using random train/test splits, with little attention to uncertainty. Random splitting can overestimate performance when data have spatial structure.

Our study differs. We use spatial cross-validation instead of random splits, and we quantify prediction uncertainty. Direct R^2^ comparisons across studies are difficult due to differences in predictors, validation methods, and settings. Our aim is not a higher R^2^. Instead, we offer a framework that admits its limitations, quantifies uncertainty, and tests spatial generalization.

**Table 6:** Comparison of selected machine learning studies for isotope prediction, including modeling strategy, predictors, and validation/uncertainty approaches.

Study	Region / context	Target	ML methods compared	Best model	*R*^2^ (δ^18^O)	Key inputs	Validation / uncertainty approach
Al Maliki et al.^25^	Iraq (precipitation)	Precipitation δ^18^O, δ^2^H	SVM, GBR, ANN, CatBoost, XGBoost, RF	RF	0.9	Meteorological + elevation	Train/test split (80/20); no explicit uncertainty quantification
Wang et al.^28^	China (precipitation)	Precipitation δ^18^O	XGBoost, MLP, SVM	XGBoost	0.71	21 environmental variables	Train/validation/test split + 5-fold cross-validation; no explicit uncertainty intervals
Benaafi et al.^29^	Eastern Saudi Arabia (coastal groundwater)	Groundwater δ^18^O	KNN, SVR, RF, ET, Bagging, AdaBoost, GBR, CatBoost, Stacking	Stacking	0.99	Hydrochemical ions	Model comparison; uncertainty analysis not explicitly reported
Mohallel et al.^30^	Egypt’s Eastern Desert (groundwater)	Groundwater δ^18^O	SVM	SVM	0.92	Hydrochemical ions	Train/test split (90/10); no formal uncertainty assessment
This study	the western desert margins of the mid-Nile Valley (groundwater)	Groundwater δ^18^O	LR, RF, GBR	RF	0.83	Hydrochemical + spatial variables	Spatial CV + prediction intervals

### Limitations and model constraints

This study has several limitations.

**Data imbalance.** The dataset is concentrated in the − 3‰ to -1‰ range (*n* = 54). Only 7 Miocene end-members (δ^18^O ≈ -10.5‰), 6 enriched samples (δ^18^O > 0‰), and only 2 samples bridging the − 8‰ to -5‰ gap are available. The model reliably predicts intermediate δ^18^O values but should not be used for end-member prediction.

**Spatial generalization.** Spatial cross-validation (R^2^ = 0.50) shows that a significant portion of the predictive skill from random splitting (R^2^ = 0.83) comes from spatial autocorrelation. Model evaluation based solely on random splits or LOOCV overestimates generalization capability.

**Spatial vs. chemical controls.** Longitude is the dominant predictor (importance = 0.56), indicating that the model partially learns the spatial gradient rather than fully transferable hydrochemical relationships. However, the sensitivity test without longitude (RFR R^2^ = 0.405) confirms that hydrochemical variables independently explain substantial variance.

**Transferability.** The model is calibrated to the specific hydrogeochemical setting of the mid-Nile Valley. Transferability to other aquifer systems requires site-specific recalibration.

**Future work.** Future studies should focus on collecting additional end-member and transitional samples to bridge the observed isotopic gap. Expanding the dataset would reduce prediction uncertainty and improve spatial generalization.

## Conclusions

We introduced an ensemble modeling framework to predict groundwater δ^18^O in the mid-Nile Valley using hydrochemical and spatial predictors.

Random Forest performed best under spatial cross-validation (R^2^ = 0.50), outperforming Lasso (0.45) and Gradient Boosting (0.41). Longitude, chloride, and magnesium were the main predictors consistent with hydrochemical evidence from Piper diagrams and saturation indices.

Within the data-rich range (-3‰ to -1‰, *n* = 54), the model achieved a mean absolute residual of 0.70‰ and a 95% prediction interval of ± 2.26‰. Outside this range, for Eocene end-members (*n* = 7) and enriched samples (*n* ≈ 6), uncertainty increased sharply.

Spatial cross-validation gave a more realistic estimate (R^2^ = 0.50) than random splitting (R^2^ = 0.83). Relying only on random splits overestimates generalization.

Our framework is not about a high R^2^. It is about honest assessment: quantifying uncertainty, testing spatial generalization, and admitting limitations. The model works well for intermediate δ^18^O values but should not be used for rare end-members.

Future work should focus on collecting more end-member and transitional samples to bridge the isotopic gap.

## Supplementary Information

Below is the link to the electronic supplementary material.

Supplementary Material 1

## Data Availability

The data used in this study are not publicly accessible due to ongoing research but can be obtained upon request from the corresponding author.
