# Supplementary material for: Ensemble machine learning method for δ18O prediction in groundwater
Source: Sci Rep. 2026 Jun 21;16:19194. doi: 10.1038/s41598-026-57131-y (PMC13284213; doi:10.1038/s41598-026-57131-y)
Supplement: Supplementary file 1 — Supplementary Material 1 [file 41598_2026_57131_MOESM1_ESM.docx]

**Table S1**: Outlier removal sensitivity results

| **Model** | **Condition** | **Test R²** | **LOOCV R²** | **Spatial CV R²** |
| --- | --- | --- | --- | --- |
| **RFR** | Outliers removed | 0.83 | 0.82 | **0.5** |
| **RFR** | Outliers included | 0.82 | 0.77 | **0.32** |
| **Lasso** | Outliers removed | 0.81 | 0.76 | 0.45 |
| **Lasso** | Outliers included | 0.78 | 0.74 | 0.2 |
| **GBR** | Outliers removed | 0.8 | 0.7 | 0.41 |
| **GBR** | Outliers included | 0.84 | 0.75 | 0.28 |
